# New Books

**Published:** 2010-05

**Carbon Capture and Storage**

Steve Rackley

St. Louis, MO:Elsevier, 2009. 408 pp. ISBN: 978-1-85617-636-1, $150

**Compendium of Trace Metals and Marine Biota**

Ronald Eisler

St. Louis, MO:Elsevier, 2010. 1,150 pp. ISBN: 978-0-444-53439-2, $430

**Ecotoxicology**

Erik Jorgensen, ed.

St. Louis, MO:Elsevier, 2010. 402 pp. ISBN: 978-0-444-53628-0, $79.95

**Environmental Change in Siberia: Earth Observation, Field Studies and Modelling**

Heiko Balzter, ed.

New York:Springer, 2010. 300 pp. ISBN: 978-90-481-8640-2, $169

**Environanotechnology**

Maohong Fan, I, C.P. Huang, Alan E. Bland, Zhonglin Wang, Rachid Slimane, Ian G. Wright, eds.

St. Louis, MO:Elsevier, 2010. 500 pp. ISBN: 978-0-08-054820-3, $147

**Global Environmental Change: Challenges to Science and Society in Southeastern Europe**

V. Alexandrov, M.F. Gajdusek, C.G. Knight, A. Yotova, eds.

New York:Springer, 2010. 350 pp. ISBN: 978-90-481-8694-5, $169

**Human Exposure to Pollutants via Dermal Absorption and Inhalation**

Mihalis Lazaridis, Ian Colbeck, eds.

New York:Springer, 2010. 308 pp. ISBN: 978-90-481-8662-4, $1690

**Local Governments and Climate Change: Sustainable energy Planning and Implementation in Small and Medium Sized Communities**

Maryke van Staden, Francesco Musco, eds.

New York:Springer, 2010. 300 pp. ISBN: 978-1-4020-9530-6, $179

**Long-Term Ecological Research: Between Theory and Application**

F. Müller, C. Baessler, H. Schubert, S. Klotz, eds.

New York:Springer, 2010. 350 pp. ISBN: 978-90-481-8781-2, $169

**Nanoparticles in the Water Cycle: Properties, Analysis and Environmental Relevance**

Fritz H. Frimmel, R. Niessner, eds.

New York:Springer, 2010. 240 pp. ISBN: 978-3-642-10317-9, $129

**Realizing the Energy Potential of Methane Hydrate for the United States**

Committee on Assessment of the Department of Energy’s Methane Hydrate Research and Development Program: Evaluating Methane Hydrate as a Future Energy Resource

Washington, DC:National Academies Press, 2010. 150 pp. ISBN: 978-0-309-14889-4, $36

**The Architecture of Green Economic Policies**

P.K. Rao

New York:Springer, 2010. 340 pp. ISBN: 978-3-642-05107-4, $139

**UV Radiation in Global Climate Change: Measurements, Modeling and Effects on Ecosystems**

Wei Gao, Daniel L. Schmoldt, James R. Slusser, eds.

New York:Springer, 2010. 550 pp. ISBN: 978-3-642-03312-4, $269

